# Predictive factors for failure of conservative management in patients with emphysematous pyelonephritis

**DOI:** 10.1016/j.amsu.2022.103930

**Published:** 2022-06-04

**Authors:** Moez Rahoui, Yassine Ouanes, Kays Chaker, Mokhtar Bibi, Kheireddine Mourad Dali, Ahmed Sellami, Sami Ben Rhouma, Yassine Nouira

**Affiliations:** Urology Department La Rabta Hospital, Tunis, Tunisia

**Keywords:** Conservative management, Drainage, Emphysematous pyelonephritis, Nephrectomy

## Abstract

**Introduction:**

Emphysematous pyelonephritis (EPN) is a severe form of life-threatening renal infection. Conservative treatment represents the gold standard in the management of EPN, but nephrectomy remains appropriate in certain situations.

**Objective:**

The aim of our study was to report our experience in the conservative management of emphysematous pyelonephritis and to identify the predictive factors of failure of conservative treatment.

**Patients and methods:**

This is a retrospective study including all patients treated for emphysematous pyelonephritis in our department between January 2015 and December 2020. The first-line treatment was conservative based on antibiotic therapy and drainage in case of an obstructive cause. A nephrectomy was performed in case of failure of the conservative approach. Epidemiological, clinical, biological, therapeutic, and evolutionary data were collected from the patients' files. Statistical analysis was made using SPSS version 28.

**Results:**

41 patients were included in our study. The mean age was 64.4 years old [28–91] with gender ratio of 0.46 (13H/28F). Diabetes mellitus was present in 75.6% of cases. The mean presentation delay was 3.28 days (Kaiser and Fournier, 2005; Kapoor et al., 2010; Aswathaman et al., 2008; Agha et al., 2020; Huang and Tseng, 2000; Falagas et al., 2007; Dutta et al., 2007; Dutta et al., 2007; Deoraj et al., 2018 Sep; Rahim et al., 2021 Mar; Maheshwari, 2021 Jul-Sep) [1-11]. In CT scan, 21 patients had class 1 EPN, 9 had class 2 EPN, 8 had class 3 EPN and 3 had class 4 EPN. The obstructive origin was found in 24 cases. Initially, 25 patients (60.9%) presented with severe sepsis and 7 patients (17.07%) developed a septic shock. Seven patients required nephrectomy with a mean delay of 2.12 days (Kapoor et al., 2010; Aswathaman et al., 2008; Agha et al., 2020; Huang and Tseng, 2000; Falagas et al., 2007) [2-6]. Five patients with septic shock refractory to conservative treatment and two patients whose evolution was marked by the occurrence of secondary septic shock. In the univariate analysis, thrombocytopenia, initially septic shock, and the need for hemodialysis were the predictive factors of failure of conservative management in patients with emphysematous pyelonephritis.

**Conclusion:**

Emphysematous pyelonephritis is a serious condition with significant mortality. The optimal management is based on conservative treatment in most cases. However, patients requiring hemodialysis and with thrombocytopenia and initially septic shock should be considered candidates for emergency nephrectomy.

## Highlights

• Emphysematous pyelonephritis (EPN) is a severe form of life-threatening renal infection.
• CT scan allows the diagnosis to be made at an early stage.
• Conservative treatment represents the gold standard in the management of EPN.
• Nephrectomy should be reserved for extensive forms or in case of failure of conservative treatment.

## 1 Introduction

Emphysematous pyelonephritis (EPN) is a necrotic infection of the kidney characterized by the presence of gas in the renal parenchyma, excretory cavities, or perirenal spaces []. It is a serious, life-threatening condition [1]. The diagnosis should be suspected in the presence of pyelonephritis that responds poorly to treatment or is associated with signs of severity, especially in a diabetic patient [2]. Its positive diagnosis is based on imaging, in particular CT scan, which is the reference examination for determining the presence of gas. Imaging can also be used to establish a radiological classification with a prognostic value to guide the therapeutic indications [2,3]. The treatment modalities for EPN have evolved over the years, from an aggressive surgical approach to a more conservative approach that preserves the functional value of the kidney as much as possible.

The aim of our study was to report our experience in the conservative management of emphysematous pyelonephritis and to identify the predictive factors of failure of conservative treatment. This work has been reported according to SCARE 2020 criteria [4].

## 2 Methods and materials

It was a retrospective, observational study conducted in a tertiary care center. After gaining local ethics committee approval, we included all the patients who were treated for emphysematous pyelonephritis by a conservative approach in our department from January 2015 to December 2020. Relevant laboratory and imaging tests were noted, and follow-up examinations were accessed using patient records and surgical reports. Radiological exploration was based on CT scans in all patients. On the basis of CT, EPN was grouped into four classes, as follows. Class 1: gas confined to renal parenchyma; class 2: gas extending to perinephric space and confined within the Gerota's fascia; class 3: gas extending beyond the Gerota's fascia; and class 4: gas involving both kidneys or gas in a solitary functioning kidney.

Treatment was based on a conservative approach including antibiotics, ureteral drainage, and percutaneous drainage. In the failure of a conservative approach, nephrectomy was performed. The success of conservative treatment is based on the improvement of the clinical condition, biological parameters, and the reduction, or even complete disappearance, of gas from the excretory tract and/or renal parenchyma. Patients were divided into two groups: group I receiving conservative management and group II undergoing nephrectomy. Differences between the groups were analyzed using the Wilcoxon rank-sum test for continuous variables and chi-square or Fisher's exact test for categorical variables, with P < 0.05 considered to indicate statistical significance. Analysis was carried out with SPSS 28.

## 3 Results

41 patients were included in our study. Our series consisted of 28 women and 13 men. The mean age of the patients was 64.4 years, with extremes of 28 and 91 years. Of our patients, 26 were known diabetics (63.41%). EPN was a circumstance for the discovery of diabetes in five cases (12,14%). No other immunosuppressive factors were noted. Upper excretory tract obstruction was found in 24 cases (58.53%). These were urinary lithiasis in 22 cases and obstructive megaureter complicated by lithiasis in 1 case. One patient had urinary distension related to prostatic hypertrophy. The left kidney was more commonly affected than the right (29 vs 8), three patients had bilateral involvement and one had EPN of the solitary kidney. The epidemiologic characteristics of the patients and baseline risk factors at presentation are summarized in Table 1.

**Table 1:** Baseline risk factors and final outcome.

Variables	Overall	Group I	Group II	P value
Number of patients, n, %	41	34	7	
Age, years	64.4	64.7	64.3	0.724
Male/female, n	13/28	11/23	2/5	0.127
Diabetes mellitus, n, %				
Yes	31 (75.6)	26 (76.47)	5 (71.42)	0.326
No	10 (24.4)	8 (23.53)	2 (28.58)	
Urologic records, n, %				
Yes	11 (26.83)	9 (29.03)	2 (28.58)	0.624
No	30 (73.17)	25 (70,93)	5 (71.42)	
Presentation delay, days	3.28	3.12	3.74	0.092
Obstructive pyelonephritis n, %				
Yes	24(58.53)	21 (61.72)	3 (42.85)	
No	17(41.47)	13 (39.28)	4 (57.15)	0.324
EPN Class, n, %				
1	21 (51.21)	19 (55.88)	2 (28.58)	0.089
2	9 (21.95)	7 (20.58)	2 (28.58)	
3	7 (17.08)	5 (14.7)	2 (28.58)	
4	4 (9.75)	3 (8.82)	1 (14.3)	
Blood sugar Level, mmol/l	11.24	11.05	12.14	0.724
Leucocytic count	16482.34	16975.24	16.247.32	0.782
Platelet count	215.12 x 10.3	225.18 x 10.3	112.24 x 10.3	**0.012**
Hyponatremia (<120 mmol/L)				
Yes	4(9.75)	3 (8.82)	1 (14.3)	0.124
NO	37(90.25)	31 (91.18)	6 (85.7)	
Hemodialysis, n, %				
Yes	7 (17.08)	1 (2.94)	6 (85.7)	**0.024**
NO	34 (82.92)	33 (97.06)	1 (14.3)	
Initially Septic shock, n, %				
Yes	7 (17.08)	2 (5.88)	5 (71.42)	**0.031**
No	34 (82.92)	32 (94.12)	2 (28.58)	
Management, n, %				
Antibiotics alone	14 (34.15)	12 (35.29)	2 (34.15)	0.078
Ureteral drainage + antibiotics	15 (63.58)	12 (35.29)	3 (42.58)	
Nephrostomy + antibiotics	8 (19.52)	7 (20.58)	1 (14.3)	
Percutaneous drainage + antibiotics	3 (7.32)	2 (5.88)	1 (14.33)	
bladder drainage + antibiotics	1 (2.43)	1 (2.94)	0 (0)	
Death, n, %				
Yes	3 (7.89)	1 (2.94)	3 (42.58)	**0.01**
No	38 (92.11)	33 (97.06)	4 (57.42)	

(73.17. The bladder urine culture was positive in 28 cases (71.1%), polymicrobial in 6 cases (14.63%), and negative in 7 cases (17.07%). Pyloric urine sampling isolated a germ in 3 patients with a negative initial urine culture. Blood culture was positive in eight cases (19.51%). The discrepancy between the urine samples and the blood culture in the nature of the germ was noted in two cases. The germs identified were Escherichia coli in 21 cases (51.12%), Klebsiella sp. in 4 cases (9.75%), and Enterococcus sp. in 3 cases (7.31%), Staphylococcus Aureus in 2 cases (4.87%) and Proteus mirabilis in 2 cases (4.87) (Fig. 1). In 15 cases (36.58%), the bacteria were ESBL producers. The observed resistances were 51% with amoxicillin, 37% with amoxicillin + clavulanic acid, 18% with third-generation cephalosporin, 42% with quinolones, and 39% with cotrimoxazole. In CT scan, 21 patients had class 1 EPN, 9 had class 2 EPN, 7 had class 3 EPN and 4 had class 4 EPN. Initially, 25 patients (60.9%) presented with severe sepsis and 7 patients (17.07%) developed a septic shock.

**Fig. 1:**
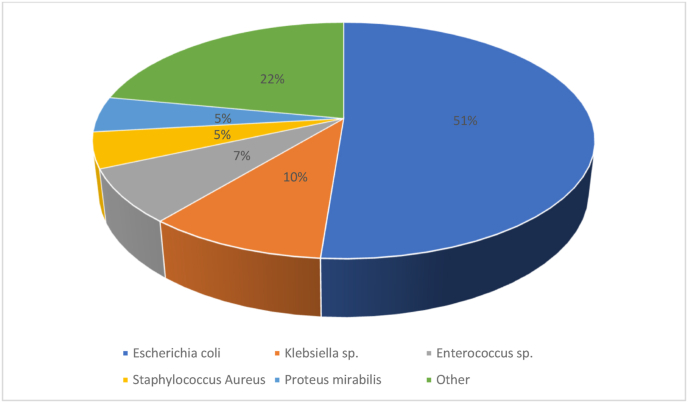
Distribution of germs isolated in bacteriological samples.

Initial treatment was based on antibiotic therapy alone in 14 cases (34,15%), ureteral drainage with antibiotic therapy in 15 cases (36,58%), percutaneous nephrostomy and antibiotic therapy in 8 cases (19,52%), bladder drainage via a suprapubic catheter and antibiotic therapy in one case (2,43%), and percutaneous drainage and antibiotic therapy in three cases (7,32%). Seven patients required nephrectomy with a mean delay of 2.12 days [[2], [3], [4], [5], [6]]. Five patients with septic shock refractory to conservative treatment and two patients whose evolution was marked by the occurrence of secondary septic shock. After nephrectomy, three patients died of multi-visceral failure. The outcome in the patients with EPN, based on CT classification and methods of management, are also given in Table 1. In the univariate analysis, thrombocytopenia, initially, septic shock and the need for hemodialysis were the predictive factors of failure of conservative management in patients with emphysematous pyelonephritis.

## 4 Discussion

PNE is a rare condition, but its incidence has been increasing since the diffusion of CT scans, which allow for more sensitive objectification of gas [1]. The average age of patients at diagnosis is 55 years with a predominantly female [2,3]. Our series includes 28 women and 13 men with a sex ratio of 0.46, the average age was 64.4 years. Bilateral forms are rare (5–20%) and are particularly severe (20 times higher mortality) [5]. The most common etiology is diabetes, which is poorly controlled and is found in 85–96% of cases [2,5]. This may be due to chronic hyperglycemia which promotes microangiopathy, anatomical and functional abnormalities of the urinary tract, and abnormalities of antibacterial immunity. Diabetic neuropathy delays diagnosis by reducing painful symptomatology and favors the occurrence of severe forms [5]. In 15% of cases, EPN is a circumstance for the discovery of diabetes [1,2,5]. The second etiological factor is the existence of a urinary tract obstruction (20–41% of cases) [2,3,6]. In our series, 75.6% of patients were diabetic and urinary tract obstruction was found in 58.53% of cases. The pathophysiology of EPN is still unclear. Currently, several authors consider that three conditions are necessary for the development of EPN [2,3,6]: the presence of gas-producing bacteria; high levels of renal tissue glucose serving as a substrate for the bacteria to produce gas; and decreased tissue perfusion. The main hypothesis is that intra-renal fermentation of glucose in the presence of facultatively anaerobic Gram-negative germs in a favorable tissue environment [7]. The gas is first formed around the papilla where the vascularization is poor, then it passes into the renal pelvis and flows along with the pyramids and into the perinephric space.

The clinical presentation of emphysematous pyelonephritis is not specific [2,5,7]. The signs are those of severe acute pyelonephritis. The features that should attract attention are the abruptness of the symptoms, occurring in a diabetic patient with a recent deterioration in general condition, possibly associated with a tendency to cardiovascular collapse [8]. Altered consciousness can be a revealing sign [2,6]. It is explained either by a severe infectious state, with consequent neurological failure, or by acid-keto compensation. A pneumaturia may be observed in cases of associated emphysematous cystitis [5,6]. The palpation of a crepitation of the lumbar fossa, in case of diagnostic delay, is exceptional.

Biological examinations confirm sepsis and look for diabetic decompensation and severity factors in the form of visceral failure (renal and liver failure) [6,7]. The majority of causative organisms are gram-negative bacilli and are indistinguishable from the strains found in normal pyelonephritis. Urine culture and/or blood cultures may isolate E. coli (60%) and Klebsiella pneumoniae (25%). The Other pathogens are less common (Proteus, Pseudomonas, Citrobacter, Acinetobacter) [2,7,8]. Anaerobic organisms are rarely isolated [9]. In 5–20% of cases, the infection is multi-microbial [3,5]. However, in 15% of cases of ENP, no infectious agent has been identified [9]. The radiological investigation is the key to the positive diagnosis of EPN. The purpose of a radiological investigation is: to confirm the diagnosis of EPN, to search for an obstructive cause in the excretory tract, and to assess the extent of infectious lesions [5,8]. Imaging is also used in the follow-up of patients and in the consolidation phase to assess possible renal sequelae.

Standard radiography can reveal renal emphysema or retro-pneumoperitoneum and can detect radiopaque lithiasis obstacles [5,10]. The sensitivity of this examination is only about 30% and areal images around and/or over the renal shadow are difficult to differentiate from the gas of intestinal origin [4,7,9]. Renal ultrasound is a difficult test to interpret in EPN. It may show hyperechogenic areas with posterior attenuation corresponding to gas bubbles [5,7,8]. It also looks for obstruction of the excretory tract. CT scan is the reference examination for the positive diagnosis, etiology, and follow-up of emphysematous pyelonephritis [2,6,11]. It is sensitive (100%) in detecting the presence of gas in the renal parenchyma and in assessing parenchymal destruction. It also allows the study of the perirenal spaces and thus specifies the extension of the lesions. The injection of contrast medium is not essential, especially as it runs the risk of acute renal failure in these patients.

Emphysematous pyelonephritis is a medical and surgical emergency. Treatment is either conservative, based on reanimation with appropriate antibiotic therapy more or less associated with drainage of the pyelocaliceal cavities or a collection, or non-conservative, based on nephrectomy [2,7,8,11]. Symptomatic treatment is carried out in an intensive care unit and consists of correction of hemodynamic, hydro electrolytic, and blood sugar disorders [7,9]. Initial probabilistic antibiotic therapy combines a third-generation cephalosporin or imipenem with a fluoroquinolone or an aminoglycoside and is then adjusted secondarily according to bacteriological findings and clinical efficacy [2,6,7,9]. Although medical treatment alone may be effective in some cases, recent studies have reported average mortality of 44%. Surgical intervention, either percutaneous or endoscopic, is most often required [7,11]. Hudson et al. first described the effective conservative treatment of EPN by percutaneous drainage in 1986 [12]. Since then, percutaneous drainage has continued to prove itself [12]. Percutaneous drainage is aimed at renal and perirenal gas and purulent collections [7,9]. Percutaneous drainage has the advantage of treating the infection site rapidly and minimally invasively while preserving the kidney. Its disadvantage may be insufficient drainage with the persistence of the septic process. In the case of peri-renal collections, or collections that spread into the retroperitoneal space, which are difficult to access by percutaneous drainage, surgical drainage can be carried out and, in most studies, avoid a first nephrectomy, which is considered abusive [8,10].

In localized forms, or in forms in which there is purulent retention secondary to a lithiasis obstacle in the excretory tract, drainage of the pyelocaliceal cavities is indicated [2,6,8]. It should be performed in a patient with a good general condition and in the absence of poor prognostic factors [2,3,6]. Urine drainage is also indicated when renal function is at risk (single kidney, bilateral pyelonephritis and chronic renal failure) or if there is a contraindication to surgery [7,8]. This drainage appears to improve the prognosis, even if there is no obvious obstruction, and should be done preferably by ureteral catheterization rather than by nephrostomy [7,9]. Simple ureteral catheter drainage or JJ is a minimally invasive means of drainage of the excretory cavities, and nephrostomy tube drainage is still indicated after failure of internal drainage. Conservative treatment requires careful monitoring in an intensive care unit and a follow-up CT scan should be performed 4–7 days after the initial drainage, or earlier if the course is poor. Currently, the indications for nephrectomy are increasingly limited to severe forms [7,9,10]. The so-called primary salvage nephrectomy is used for extensive forms with several organ dysfunctions, or secondary after the failure of conservative treatment [2,3,6,12]. Mortality after primary nephrectomy is higher than mortality after secondary nephrectomy after the failure of conservative treatment (23% versus 12.5% respectively) [2,7,12]. Conservative management should therefore be the first step in a graded treatment approach, based on primary conservative treatment (percutaneous drainage or urinary drainage), followed by secondary nephrectomy in case of failure.

Lu et al. studied the predictive factors for failure of conservative treatment in 43 patients [13]. The failure rate of conservative management was 32.6% (14/43). Severe hypoalbuminemia (p = 0.003), use of emergency hemodialysis (p = 0.03), and polymicrobial infections (p = 0.04) were significantly associated with failure of conservative treatment. EPN is characterized by a severe prognosis. It is a severe infection with spontaneous mortality without treatment of 100%. In treated patients, the mortality rate varies from 12% to 47%, even in tertiary care settings [7,11,10,13]. According to Wan et al. [14], the prognosis is worse when there is significant parenchymal destruction, renal insufficiency (creatinine level above 120 mmol/L), thrombocytopenia (below 60,000 cells/mm3), and hematuria, the extent of which reflects the severity of renal destruction and/or the presence of venous thrombosis [14]. According to Lu et al. [13], the need for emergency hemodialysis, septic shock, altered consciousness, severe hypoalbuminemia, inappropriate antibiotic therapy, and polymicrobial infection were prognostic factors significantly associated with higher mortality. In our study, the failure rate of conservative treatment was 17.07%. In the univariate analysis, thrombocytopenia, initially septic shock, and the need for hemodialysis were the predictive factors of failure of conservative management in patients with emphysematous pyelonephritis.

The prognosis of long-term renal function depends on the degree of parenchymal destruction and the existence of an associated renal disease. Hence the importance of conservative treatment whenever possible, particularly in diabetic patients.

Several limitations of this study must be acknowledged before interpreting our findings. First, this study was based on a limited population at a single institution. Second, the retrospective descriptive design was not ideal for attaining study goals. Despite these limitations, this study demonstrated that thrombocytopenia, initially septic shock, and the need for hemodialysis were the predictive factors of failure of conservative management in patients with emphysematous pyelonephritis.

## 5 Conclusion

EPN remains a serious infection with a life-threatening and functional prognosis. CT scans allow the diagnosis to be made at an early stage. The therapeutic attitude is currently conservative, based on reanimation measures, early adapted antibiotherapy in addition to percutaneous drainage of peri-renal collections and drainage of the urinary tract in case of obstruction. Nephrectomy should be reserved for extensive forms with several organ dysfunctions or in case of failure of conservative treatment.

## Ethical approval

Not applicable.

## Funding

We have any financial sources for our research.

## Author contributions

Rahoui Moez, Yassine ouannes and Kays chaker: Data collection, Manuscript writing, Results discussion. Bibi Mokhtar, Kheireddine Mourad Dali and Ahmed sellami: Manuscript writing and revision. Ben rhouma sami and Nouira yassine: Paper revision.

Provenance and peer review.

## Registration of research studies

1. Name of the registry:: N/a.

2. Unique identifying number or registration ID:: N/a.

3. Hyperlink to your specific registration (must be publicly accessible and will be checked):: N/a.

## Guarantor

Rahoui Moez is the guarantor of the study and accept full responsibility for the work and/or the conduct of the study, had access to the data and controlled the decision to publish.

Not commissioned, externally peer-reviewed.

## Consent

Written informed consent was obtained from the patient for publication of this case report and accompanying images. A copy of the written consent is available for review by the Editor-in-Chief of this journal on request.

## Declaration of competing interest

Authors do not report any conflict of interest.
